# Effects of collaborative care on recognition and management of common mental disorders by general practitioners: a cluster-randomised trial in Norway

**DOI:** 10.1186/s12875-026-03227-3

**Published:** 2026-02-26

**Authors:** Torleif Ruud, Jūratė Šaltytė Benth, Ajmal Hussain, Jorun Rugkåsa, Mina Piiksi Dahli, Ketil Hanssen-Bauer, Mette Brekke, Nick Kates, Ole Gunnar Tveit, Inger Cathrine Kann, Ole Rikard Haavet

**Affiliations:** 1 Division of Mental Health and Addiction Services, Akershus University Hospital, Lørenskog, Norway; 2 Institute for Clinical Medicine, Medical Faculty, University of Oslo, Oslo, Norway; 3 Health Services Research Unit, Akershus University Hospital, Lørenskog, Norway; 4 Faculty of Health Sciences, Oslo Metropolitan University, Oslo, Norway; 5 Department of General Practice, Institute of Health and Society, Faculty of Medicine, University of Oslo, Oslo, Norway; 6 Department of Psychiatry, McMaster University, Hamilton, ON Canada; 7 Norwegian Labour and Welfare Administration, Oslo, Norway

**Keywords:** Collaborative care, General practice, Primary care, Mental health services, Cluster-randomised controlled trial

## Abstract

**Background:**

The aim of the study was to determine the effects on health care of an adapted Norwegian version of a Canadian model of collaborative care, involving general practitioners (GPs) and mental health specialists working together co-located in GP practices. In previous papers, we have shown that the adapted model was successfully implemented and found to be beneficial by participating GPs, improving their detection of anxiety in young people, and with a reduction in long term sickness benefits. The current study examines whether collaborative care was associated with changes in (a) the number of referrals from GPs to mental health services, (b) the number of GP patients provided outpatient visits in mental health services, (c) GPs’ recognition of common mental disorders, and (d) GPs’ prescription of various types of psychotropic medication.

**Methods:**

The study was a cluster-randomised controlled trial of the collaborative care model in three GP practices (intervention group) compared with usual health care in three other practices (control group) in Oslo, Norway. A clinical psychologist and a psychiatrist from a community mental health centre worked half time and two hours per week, respectively, in each intervention practice for 18 months. They were available for case discussions and provided assessments and brief therapies. Structured data were extracted retrospectively from the electronic patient records of both GPs and mental health services for 12 months before and during the implementation of collaborative care for patients 16–65 years old. Data were analysed with generalized linear mixed models.

**Results:**

There were no significant differences in referrals to mental health services (the primary outcome) and in the use of outpatient specialised mental health services. The GPs in the intervention practices diagnosed significantly more patients with common mental disorders (anxiety, depression), and these changes were significantly associated with a reduction in unexplained physical symptoms. Significant changes in prescribing patterns of psychotropic medication were consistent with the increased recognition of mental disorders, and their use was possibly more appropriate.

**Conclusions:**

Collaborative care with co-located mental health specialists in GP practices led to an increased recognition of common mental disorders by those GPs. Due to a lack of structured clinical measurements in the electronic patient records, the clinical outcomes of the intervention were unknown.

**Supplementary Information:**

The online version contains supplementary material available at 10.1186/s12875-026-03227-3.

## Background

In Norway, as elsewhere, general practitioners (GPs) play a central role in the delivery of both general and mental health care [1]. They see most patients with mental problems or disorders, and more than mental health services [2–4]. This makes their role in recognising and diagnosing common mental disorders, such as depression and anxiety as well as a range of other mental health problems, vital if patients are to receive the care they need, including referrals to mental health services. GPs provide various types of treatment and support for mental health problems, including psychotropic medication when this is indicated. GPs also write the majority of prescriptions for psychotropic medications for Norwegian patients, significantly more than psychiatrists and other groups of physicians [5], and they initiate the majority of prescriptions for treatment of anxiety and depression [6].

However, for GPs who have busy schedules and multiple responsibilities [7], diagnosing mental disorders among the wide range of health problems they see can be challenging [8–12]. GPs only recognise a proportion of mental health problems, but recognition increases with symptom severity [8, 13]. Anxiety or depression are more likely to be recognized when GPs are more confident in their abilities to identify depression [14].

Collaboration between GPs and mental health services has increasingly been recognised as important for the overall wellbeing of patients, and collaborative care models have been shown to increase the amount of mental health care provided in GP practices [15]. Reviews of studies of several models of collaborative care have shown some positive and some non-significant findings [16–22]. Position papers across different models and countries have defined and discussed principles for collaborative care and how these can be applied in various circumstances and contexts [23, 24].

The Hamilton Family Health Teams (HFHT) model developed in Hamilton, Canada, is a well-established model of collaborative care that brings mental health counsellors and psychiatrists into GP practices, creating mental health teams involving the GPs, the practice nurse(s), the mental health counsellors and the visiting consultant psychiatrist [15, 25]. The model has increased access to care for persons with mental health problems, reduced waiting times for service, reduced referrals to outpatient mental health services, increased patient satisfaction, improved patient health, improved communication and co-ordination of care, and increased GPs’ confidence and skills in recognising and treating mental health problems [15, 25].

Norway has also seen the development of various models of collaboration between community mental health centres (CMHCs), GPs and primary health and social care. But with a lack of research on such models, there is little knowledge as to whether they contribute to better health care for the large group of patients with common mental health problems seen by GPs. Inspired by the HFHT model, we developed an adapted model customised as a close collaboration between participating GP practices and mental health services. The development and implementation of the model have been described and evaluated in a previous publication [26]. In a qualitative study, the GPs who participated in this collaborative care model identified that co-location with frontline mental health specialists was a key factor in the model’s success [27]. The model has been shown to result in a reduction in long term sickness benefits [28] and to improve GPs’ detection of anxiety in young people [29]. However, a final publication on the model’s clinical impact on GPs assessment and management of mental health problems has not yet been published.

### Aims

The aim of the current paper was to determine the effects on health care of the Norwegian adaption of a Canadian model of collaborative care provided by co-located GPs and mental health specialists. The research questions were whether collaborative care was associated with changes in (a) the number of referrals made by GPs to mental health services, (b) number of GP patients provided outpatient in mental health services, (c) GPs’ recognition of common mental disorders (anxiety and depression), and (d) GPs’ prescription of various types of psychotropic medication.

## Methods

### Design

The study was a cluster-randomised controlled trial (CRCT) that examined the effects of collaborative care on patient health care in comparison to usual health care. The trial took place across three Oslo boroughs, and we recruited two GP practices in each borough. These were randomised so that one GP practice in each borough was assigned to the collaborative care model for 18 months (intervention group), and the other to provide usual healthcare (control group). Data on all GPs’ patient contacts were retrospectively extracted from electronic patient records at both the intervention and control practices. These data covered a 12-month period prior to the start of the collaborative care initiative (the 2015 cohort) and the final 12 months of the 18-month trial period (the 2017 cohort). Figure 1 in the Supplementary Material shows the design. The effects of the model were assessed by comparing outcomes of interest, analysing the differences between the intervention and control groups across the two cohorts. The trial followed the CONSORT statement guidelines for parallel groups randomised trials [30, 31]. The trial was registered retrospectively in ClinicalTrials.gov (identifier NCT03624829, registered August 5th, 2018).

### Context

Most health care in Norway is publicly funded [32, 33]. Municipalities are responsible for organizing and providing primary health care, including GP services, local mental health care, and local substance abuse care. Specialised mental health services (henceforth called mental health services) and general hospitals are funded by health trusts, owned by four regional health authorities. This division in funding pathway for primary care and specialised health services contributes to divergent tasks and priorities, complicating coordination and collaboration across these sectors. CMHCs are mandated to collaborate with GPs and be available for clinical guidance and advisory support, but there is limited knowledge about the extent and effects of such collaborations.

Based on a patient-list system for GPs, all Norwegians have a right to health care from a regular GP of their choice, and 99% of the population was registered with a regular GP in January 2017 [34]. The average GP list size was 1,120 persons in 2016 [35]. The majority of GPs in Norway are self-employed and reimbursed through a mixed model: approximately 35% from municipal funding, based on the number of patients on their list, 35% via fee-for-service reimbursements from the national insurance scheme according to patient contacts reported with codes from the International Classification of Primary Care version 2 (ICPC-2), and 30% from patient co-payments [35]. A small but increasing number of GPs are employed by the municipality, with regular salaries.

The study was conducted in three Oslo boroughs (Alna, Grorud, Stovner) with a total population of 108 000. Health services in the area included 85 GPs in 20 GP practices, primary health and social care provided by the boroughs, and mental health services provided by a CMHC, child and adolescent mental health services (CAMHS) and other departments at the health trust Akershus University Hospital.

### The intervention model of collaborative care

The intervention was an adapted version of the Canadian HFHT model. The core component consisted of a clinical consultant psychologist from the local CMHC working half time and a psychiatrist working two hours a week, co-located in each of the three intervention GP practices for a period of 18 months. The psychologist and the psychiatrist were available to the GPs for consultation, case discussions, selected joint consultations and assessments, and brief therapy for patients, if requested to do so by the GP. Other mental health or substance addiction services were involved or consulted according to need. An extended component of the intervention consisted of collaboration between the co-located team and other primary care health and social services in the borough. The adapted model and its implementation is described in detail in a previous paper [26].

Collaborative care is a complex intervention. We hypothesised that the model’s mechanism for change was the close interaction between co-located GPs and mental health specialists. Their different expertise would facilitate building and utilising relationships, learning from each other when working together, and providing more comprehensive assessments and treatments for patients with mental health problems [26]. For GPs, this might lead to improved recognition and management of common mental disorders.

The decisions on the adaption of the model were taken by the collaborating services themselves in 2015 after a visit to Hamilton by a group of 14 people involved with the project, and with input from the research group. Some further adaptations of the model to the local context were made at each of the three intervention practices. The collaborative care in the three intervention GP practices was established during the winter of 2016 and was operational for 18 months from April/May 2016 to October/November 2017 [26].

### Recruitment and randomisation of GP practices

Using availability sampling, medium-sized GP practices (4–6 GPs) were invited to participate in the CRCT. Each practice was contacted by phone, followed by a visit from a research group GP and psychiatrist to explain the project. The practice’s GPs then decided on participation and signed a consent agreement, including consent to randomisation. Nine practices were approached until six had agreed to participate. Stratifying by boroughs, a statistician randomised the two practices in each borough to either the intervention (collaborative care) or control (usual care) group.

### Patient samples

The 2015 cohort included all patients 16–65 years old who had had contact with the six GP practices during the 12 months before randomization. The 2017 cohort included all patients 16–65 years old who had contact with the six GP practices during the 12 last months of collaborative care after a six-month start-up phase of the collaborative care. There were no exclusion criteria. Based on previous studies we estimated that each cohort could include 20,000 patients.

Table 1 shows the number of GPs and the number, sex and age of the patients in the intervention and control practices of the 2015 cohort and the 2017 cohort. The randomisation of practices led to 10 GPs in the intervention group and 20 GPs in the control group. With a similar average number of list patients per GP, this resulted in a patient sample in the control group being twice as large as the patient sample in the intervention group. There are some small significant differences between the intervention and control groups in patients’ age and sex. Difference between sexes were not significant among intervention practices (*p* = 0.655). Among the control practices, there were small between-sex differences between the cohorts (*p* = 0.039). Mean age differed significantly between the cohorts (*p* < 0.001 and *p* = 0.019 in the intervention and control group, respectively).

**Table 1:** GP practices and GP patients’ characteristics in the cluster-randomised controlled trial

Cohort and characteristics	Intervention practices	Control practices	*p*-value of difference
GP practices			
Number of GP practices	3	3	
Number of GPs	10	20	
Patient cohort 2015	6,093	12,532	
Sex, n (%)			
Female	3,471 (57.0)	6,946 (55.4)	0.047^a^
Male	2,622 (43.0)	5,586 (44.6)	
Age, mean (SD)	41.0 (14.1)	39.7 (13.5)	< 0.001^b^
Age groups, n (%)			
16–19	483 (7.9)	942 (7.5)	
20–29	1,094 (18.0)	2,436 (19.4)	
30–39	1,162 (19.1)	2,906 (23.2)	
40–49	1,358 (22.3)	2,871 (22.9)	
50–59	1,350 (22.2)	2,266 (18.1)	
60–65	646 (10.6)	1,111 (8.9)	
Patient cohort 2017	6,429	12,774	
Sex, n (%)			
Female	3,637 (56.6)	6,915 (54.1)	0.001^a^
Male	2,792 (43.4)	5,859 (45.9)	
Age, mean (SD)	39.7 (14.2)	39.3 (13.6)	0.058^b^
Age groups, n (%)			
16–19	608 (9.5)	1,046 (8.2)	
20–29	1,266 (19.7)	2,441 (19.1)	
30–39	1,327 (20.6)	3,037 (23.8)	
40–49	1,283 (20.0)	2,889 (22.6)	
50–59	1,376 (21.4)	2,443 (19.1)	
60–65	569 (8.9)	918 0.2)	

Statistical tests: ^a^χ^2^-test, ^b^Two-sided independent samples t-test

### Measures

#### Outcome measures

Because we used structured outcome data from electronic patient records, we were limited to the use of intermediate outcomes measuring elements of health care as specified below [36]. The electronic patient records did not contain structured measures of clinical outcomes for common mental disorders, and we did not have approval to extract text from the electronic records.

The primary outcome measure for this study was the number of referrals from GPs to the mental health services, with referrals as the unit of analysis. All referrals were assessed by intake teams to determine whether the referred patient was eligible for mental health care according to guidelines from the national health authority, and this was registered in the electronic patient records. We used the percentage of referrals from GPs that were found to be eligible in the mental health services as a secondary measure of the appropriateness and quality of the referrals. Referrals within the health trust were not included in our trial.

We had three other secondary outcome measures, with the individual patient as the unit of analysis. The first was the number of patients of these GPs being seen for one or more outpatient consultations or visits at the mental health services at Akershus University Hospital, and the average number of visits that each patient received.

The second was the number of patients given an ICPC-2 code of a mental health problem by their GP at least once during the 12 months. These codes were registered in the electronic patient records, as the GPs were required to register one or more ICPC-2 code at each consultation or visit to receive reimbursement from the Norwegian Health Economics Administration [37]. ICPC-2 includes two types of codes: diagnostic codes for specific illnesses, and codes for symptoms or complaints when a diagnostic code for an illness cannot be established. Most of the collaborative care research in mental health has focused on depression and anxiety, given their high prevalence. We also focused on these conditions, grouped as anxiety (including the diagnostic codes P72 anxiety disorder and P79 phobia/compulsive disorder), depression (the diagnostic code P76 depressive disorder), and common mental disorder (any of these three diagnostic codes). Codes for other types of mental health issues were not included in our data analyses.

The third secondary outcome was the number of patients who received a prescription from their GP for a psychotropic medication at least once during the 12 months. We divided the psychotropic medications into five pharmacological subgroups based on ATC-codes (The Anatomical Therapeutic Chemical Classification System) [38]. The subgroups (with the ATC code for each) were antipsychotics (N05A), antidepressants (N06A), anxiolytics (N05B), hypnotics/sedatives (N05C), and central stimulants (N06B).

#### Explanatory variables

Explanatory variables included age, sex, and ICPC-2 codes for illnesses or symptoms/complaints other than mental health illnesses and problems. We hypothesised that an increasing recognition of mental health problems – either experienced or presented as physical symptoms - would be associated with a decreased utilisation by GPs of codes for unexplained physical symptoms. Two experienced GPs in our research group identified a substantial number of such unexplained physical symptom codes, such as tiredness, headache, abdominal pain and unspecified musculoskeletal disorder symptoms. We grouped these as ‘symptom codes’. All other codes for physical illness or complaints were grouped as ‘other codes’.

### Data collection

Structured data from the electronic patient records at the GP practices was extracted retrospectively in 2015 for the 2015 cohort (12 months) and in 2017 for the 2017 cohort (the last 12 months of the 18 months intervention period, excluding data from the first 6 months when the collaborative care model was being established). Data extracted for each contact were patient ID, age, sex, date of contact, ICPC-2 codes, and any medication prescription. The data extraction from the GP practices was conducted by the firm Mediata AS, using their software for extracting data from the various systems of electronic patient records that were used in Norwegian GP practices. Data on referrals and outpatient visits in the mental health services for the same two patient cohorts were extracted from the electronic patient records at Akershus University Hospital in 2017 by the data management department at the hospital.

### Data analyses

We reported on the number of GPs, patients and patient characteristics (age and sex) in each cohort and in each group (intervention and control) of GP practices. Between-cohort differences in sex and age within each practice group were assessed by x^2^-test or z-test for proportions and independent samples t-test, respectively.

Outcomes and patient characteristics were presented as means and standard deviations (SDs) for continuous data, and as frequencies and percentages for categorical data. Due to multiple records for some patients and the study design, a cluster effect was expected to be present in the data. As quantified by an intra-class correlation coefficient for all outcome variables, the cluster effect at the GP practice level was weak or non-existent, and therefore not adjusted for in the analyses. Between-group differences in referrals, diagnoses, and prescriptions for the 2015 and 2017 cohorts were therefore assessed by generalized linear mixed models with logit link function, random effects for patients and fixed effects for group (intervention vs. control), cohort (2015 vs. 2017), and an interaction between the group and cohort. The models were further adjusted for the age and sex of the patient.

For diagnoses, the models were also adjusted for ‘symptom codes’ and ‘other codes’, which were included as additional fixed effects together with the interaction between the diagnosis and cohort. Bayes Information Criterion (BIC) was applied to eliminate potentially excessive interactions from the model. For prescriptions, the model was additionally adjusted for time (days), as there could potentially be a trend in prescription rates with time. Since all the models contained interactions between group and cohort, the results were tabulated as regression coefficients (RCs) and standard errors (SEs) instead of odds ratios (ORs) and confidence intervals (CIs) and made available as Supplementary Material. To make interpretation easier, post hoc analyses were performed, and the results were tabulated as ORs or means with corresponding 95% CIs for within- and between-cohort differences. Results with p-values below 0.05 were considered statistically significant. All statistical analyses were performed in STATA version 17.

## Results

### Number of referrals to mental health services

The results for referrals of patients to mental health services are shown in Table 2 and Supplementary Table 2B in the Supplementary Material. There was no significant difference between the intervention and control practices in changes in the odds of being referred to the mental health services. In 2015 there were significantly lower odds in the intervention group than in the control group, but not in 2017. No significant differences were found when stratifying the analyses by inpatients/outpatients or by CAMHS/departments for adults/substance use departments.

**Table 2:** Change in referrals to mental health services. Descriptive statistics and results of generalized linear mixed models for differences between intervention and control practices across 2015 and 2017 cohorts

Descriptive data on number (%) of referrals				
Outcome variables	Intervention practice		Control practice	
	Cohort 2015	Cohort 2017	Cohort 2015	Cohort 2017
GP patients with extern referrals to mental health services overall				
No	5,900 (97.9)	6,165 (97.7)	12,002 (97.4)	12,314 (97.7)
Yes	125 (2.1)	144 (2.3)	326 (2.6)	292 (2.3)
GP patients with extern referrals to type of mental health services				
No	5,900 (97.9)	6,165 (97.7)	12,002 (97.4)	12,314 (97.7)
Yes, outpatient service	100 (1.7)	116 (1.8)	256 (2.1)	251 (2.0)
Yes, inpatient service	25 (0.4)	28 (0.4)	70 (0.6)	41 (0.3)
GP patients with extern referrals to mental health services departments				
No	5,900 (97.9)	6,165 (97.7)	12,002 (97.4)	12,314 (97.7)
Yes, CAMHS	12 (0.2)	11 (0.2)	13 (0.1)	21 (0.2)
Yes, mental health services adults	102 (1.7)	120 (1.9)	276 (2.2)	233 (1.9)
Yes, substance use department	11 (0.2)	13 (0.2)	276 (2.2)	38 (0.30)

Results of generalized linear mixed model for differences between intervention and control practices in change in external referrals to specialised mental health services (RC=regression coefficient, SE=standard error)		
	RC (SE)	*p*-value
Constant	−5.78 (0.23)	<0.001
Cohort	−0.23 (0.11)	0.032
Group of practices	−0.35 (0.16)	0.025
Cohort x Group	0.25 (0.20)	0.192
Patient age	−0.02 (0.004)	<0.001
Patient sex (male)	0.24 (0.11)	0.031

Another possible change in referrals patterns could have been due to a shift in the proportion of referrals accepted or rejected by the mental health services’ intake teams. There was a reduction in rejections of external referrals from 13 (10%) in 2015 to none in 2017 for the intervention group, and from 17 (5%) to 1 for the control group. This represents a substantial reduction for both groups of practices. However, statistical analysis was not feasible due to the low numbers of cases in the 2017 cohort.

### Number of patients seen as outpatients in the mental health services

The results of GP patients who had outpatient visits in the mental health services are shown in Table 3 and Supplementary Table 3B in the Supplementary Material. For adolescents (age 16–17), there was no between-group difference in the change in odds of attending outpatient mental health services, nor in the average number of visits from 2015 to 2017. For adults in the intervention group, there were significantly lower odds of being an outpatient compared to the control group for both cohorts, but with no significant difference between the cohorts (no significant interaction).

**Table 3:** Number of GP patients seen as outpatients in mental health services. Descriptive statistics and results of generalized linear mixed model for differences between intervention and control groups across cohorts in change for being outpatients in mental health services

Number (%) of GP patients with outpatient sessions at mental health services				
	Intervention practices		Control practices	
Age groups	Cohort 2015	Cohort 2017	Cohort 2015	Cohort 2017
Adolescents (age 16-17)				
No	231 (92.8)	280 (92.4)	432 (94.1)	488 (93.3)
Yes	18 (7.2)	23 (7.6)	27 (5.9)	35 (6.7)
Adults				
No	5,601 (97.0)	5,845 (97.5)	11,399 (96.0)	11,646 (96.4)
Yes	175 (3.0)	153 (2.6)	476 (4.0)	432 (3.6)

Average number (%) of visits in mental health outpatient clinics for those being outpatients				
	Intervention practices		Control practices	
Age groups	Cohort 2015	Cohort 2017	Cohort 2015	Cohort 2017
Adolescents	6.8 (5.9)	10.5 (9.9)	9.6 (7.4)	8.4 (7.9)
Adults	12.0 (13.5)	13.1 (12.9)	12.6 (13.2)	12.3 (13.9)

Results of generalized linear mixed models for differences between intervention and control practices in change in of odds for being outpatients in mental health services. Regression coefficient (RC) and standard error (SE)				
Results of generalized linear mixed model for adolescent outpatients in mental health services				
	RC (SE)	*p*-value	RC (SE)	*p*-value
Constant	-1.32 (3.39)	0.697	39.05 (25.85)	0.131
Cohort	0.14 (0.27)	0.598	-0.88 (2.00)	0.662
Practice group	0.21 (0.32)	0.502	-2.77 (2.37)	0.244
Cohort x Group	-0.08 (0.42)	0.856	4.96 (3.16)	0.117
Patient age	-0.07 (0.21)	0.726	-1.78 (1.57)	0.259
Patient sex (man)	-0.61 (0.21)	0.004	-0.93 (1.65)	0.574

Results of generalized linear mixed modes for adult outpatients in mental health services				
	RC (SE)	*p*-value	RC (SE)	*p*-value
Constant	-4.41 (0.18)	<0.001	13.06 (1.40)	<0.001
Cohort	-0.19 (0.09)	0.030	-0.05 (0.84)	0.949
Practice group	-0.35 (0.13)	0.008	-0.61 (1.17)	0.601
Cohort x Group	-0.15 (0.16)	0.363	0.89 (1.65)	0.589
Patient age	-0.02 (0.004)	<0.001	-0.002 (0.03)	0.962
Patient sex (male)	-0.21 (0.09)	0.026	-1.56 (0.80)	0.051

### GPs’ recognition of common mental disorders

Table 4 and Supplementary Table 4B in the Supplementary Material present the results of the GPs recognition of mental health problems. There was a significantly higher increase in the likelihood of recognising anxiety, depression and common mental disorders overall in the intervention group compared to the control group (significant interaction terms). In the 2015 cohort, the odds for recognising anxiety, depression and common mental disorders were lower for patients in the intervention group compared to the control group, while no significant differences were noted between groups in the 2017 cohort. While there was a weak but non-significant decrease in the odds of recognising all three conditions in the control group, the intervention group showed a significant increase in this area. All three conditions were significantly associated with the reduction of ‘symptom codes’ and the increase of ‘other codes’.

**Table 4:** Patients diagnosed with a common mental disorder at least once for 2015 and 2017 cohorts. Descriptive statistics and results of generalized linear mixed models for differences between groups in change in common mental disorders

Descriptive statistics: Number (%) of patients with different groups of diagnosed problems				
Diagnosed problem	Intervention practices		Control practices	
	Cohort 2015	Cohort 2017	Cohort 2015	Cohort 2017
Common mental disorder	320 (5.9)	400 (7.2)	919 (8.1)	839 (7.4)
Anxiety	113 (2.1)	151 (2.7)	297 (2.6)	261 (2.3)
Depression	224 (4.1)	276 (4.9)	692 (6.1)	623 (5.5)
Symptom codes	2,323 (42.8)	2,328 (41.7)	5,652 (49.5)	5,720 (50.2)
Other codes	5,073 (93.4)	5,266 (94.3)	10,433 (91.4)	10,783 (92.0)

Results of generalized linear mixed models of associations between problems, group of practices and patient cohorts. Regression coefficient (RC) and standard error (SE)						
	Common mental disorder		Anxiety		Depression	
	RC (SE)	*p*-value	RC (SE)	*p*-value	RC (SE)	*p*-value
Constant	-2.53 (0.10)	<0.001	-5.83 (0.29)	<0.001	-5.07 (0.23)	<0.001
Cohort	-0.09 (0.05)	0.068	-0.19 (0.11)	0.072	-0.13 (0.07)	0.071
Practice groups	-0.33 (0.07)	<0.001	-0.30 (0.16)	0.056	-0.58 (0.12)	<0.001
Cohort x Group	0.32 (0.09)	0.001	0.63 (0.19)	0.001	0.40 (0.14)	0.005
Age	0.01 (0.002)	<0.001	0.002 (0.004)	0.695	0.02 (0.003)	<0.001
Sex (male)	-0.36 (0.04)	<0.001	-0.34 (0.11)	0.003	-0.64 (0.09)	<0.001
Symptom codes	0.28 (0.04)	<0.001	0.43 (0.11)	<0.001	0.36 (0.07)	<0.001
Other codes	-0.32 (0.07)	<0.001	-0.63 (0.17)	<0.001	-0.42 (0.13)	0.001

### GPs’ prescription of psychotropic medication

Table 5 and Supplementary Table 5B in the Supplementary Material show the results for GPs’ prescriptions of psychotropic medication. There were significant between-group differences in the change between the cohorts in the odds for prescribing antipsychotics, anxiolytics, hypnotics/sedatives, and stimulants (significant interactions). In the 2015 cohort, the control group had significantly higher odds than the intervention group for being prescribed antipsychotics and stimulants but significantly lower for antidepressants. In the 2017 cohort, the odds for hypnotics/sedatives were higher in the control group, while the odds for antidepressants were higher in the intervention group. Finally, there was a significant increase in odds for antipsychotics and a significant reduction in the odds for hypnotics/sedatives in the intervention group, while there were significant increases for stimulants in both groups.

**Table 5:** Number of patients with prescription of psychotropic drugs in intervention and control groups at least once for 2015 and 2017 cohorts. Descriptives and results of logistic regression models for differences between groups in change in number of patients with prescription

Descriptive statistics: Number (%) of patients with prescriptions of types of psychotropic medication				
Psychotropic medication group	Intervention practices		Control practices	
	Cohort 2015	Cohort 2017	Cohort 2015	Cohort 2017
Antipsychotics	335 (10.8)	392 (12.2)	1,455 (13.4)	1,050 (12.6)
Anxiolytic	746 (23.9)	755 (23.5)	3,312 (30.4)	2,334 (28.0)
Hypnotics/Sedatives	1,162 (37.3)	1,121 (34.8)	3,778 (34.7)	2,975 (35.7)
Antidepressants	839 (26.9)	856 (26.6)	2,142 (19.7)	1,717 (20.6)
Stimulants	35 (1.1)	94 (2.9)	206 (1.9)	268 (3.2)

Results of generalized linear mixed models of associations between prescriptions, group of practices and patient cohorts. Regression coefficient (RC) and standard error (SE)						
	Antipsychotics		Anxiolytics		Hypnotics / Sedatives	
	RC (SE)	*p*-value	RC (SE)	*p*-value	RC (SE)	*p*-value
Constant	-4.54 (0.31)	<0.001	-3.71 (0.25)	<0.001	-2.56 (0.23)	<0.001
Cohort	-0.04 (0.07)	0.545	-0.10 (0.05)	0.062	0.08 (0.05)	0.130
Practice group	-0.36 (0.17)	0.032	-0.21 (0.14)	0.124	-0.002 (0.13)	0.988
Cohort x Group	0.44 (0.15)	0.003	0.28 (0.11)	0.012	-0.37 (0.10)	<0.001
Age	-0.01 (0.005)	0.027	0.009 (0.005)	0.056	0.01 (0.005)	<0.001
Sex (male)	0.74 (0.13)	<0.001	-0.14 (0.12)	0.221	0.03 (0.11)	0.800
Day no.	0.0002 (0.0002)	0.312	0.0003 (0.0002)	0.051	-0.0004 (0.0002)	0.030

	Antidepressants		Stimulants	
	RC (SE)	*p*-value	RC (SE)	*p*-value
Constant	-1.47 (0.22)	<0.001	-0.05 (0.18)	0.764
Cohort	-0.02 (0.06)	0.759	0.47 (0.10)	<0.001
Practice group	0.36 (0.13)	0.006	-0.62 (0.19)	0.001
Cohort x Group	-0.10 (0.11)	0.398	0.50 (0.23)	0.027
Age	-0.007 (0.004)	0.116	-0.10 (0.004)	<0.001
Sex (male)	-0.62 (0.11)	<0.001	0.34 (0.08)	0.808
Day no.	-0.0001 (0.0002)	0.554	0.00009 (0.0004)	0.764

## Discussion

### Number of referrals to mental health services

There was no significant difference between the intervention and control groups in the change in odds of being referred to the mental health services. We had anticipated a reduction in referrals from the intervention group versus the control group. This was based on the premise that GPs would gain greater confidence in treating mental health issues themselves, and that the psychologists would provide brief therapy to patients who would otherwise have been referred. Conversely, it is plausible that the intervention simultaneously led to improved GP awareness and recognition of mental health problems, thereby increasing the number of patients assessed as requiring referral. This understanding is supported by feedback from GPs in the intervention practices and by reports from the psychologists, who identified many patients with complex needs who were subsequently referred [27], The improved detection and triage of patients requiring specialist care may therefore have counteracted the expected decrease in referrals.

A nationwide registry-based study of all adult patients with a new depression diagnosis in general practice in Norway 2009–2015 showed a weak increase in referrals to mental health care over the years [39], while a systematic review of studies of mental health workers co-located in primary care practices in four countries found an increase in referrals among minority populations [21].

It could also be that GPs in the intervention practices improved their selection of patients to be referred, and the quality of their referral letters. The increase in the proportion of referrals accepted by the mental health services intake teams might indicate such a change, but this was similar in both the intervention and the control group.

### Number of patients seen as outpatients in the mental health services

Even though the groups are small, we found it important to analyse adolescents and adults separately, because they are served by different mental health departments with which the GPs collaborate. The lack of significant changes in the odds of youth being seen as an outpatient in mental health services is consistent with the non-significant changes in the number of referrals, but the results must be interpreted cautiously.

A study of the GP patients in the 2015 cohort who were outpatients in the mental health services showed that men were more likely to be outpatients than women, which might imply that the thresholds for becoming outpatients were lower for men than women [40]. In Hamilton, the substantial increase in mental health treatment being provided in HFHT practices when compared to those being provided in outpatient mental health services had evolved over time [15]. It is possible that a similar development could have been observed in Norway if the model had been sustained and further developed over years.

### GPs’ recognition of common mental disorders

There was a significantly higher increase in the odds in the intervention group compared to the control group for recognising anxiety, depression and common mental disorders. In the 2015 cohort, the odds for recognising the three types of conditions were lower in the intervention group than the control group. This suggests that the close collaboration between GPs and co-located mental health specialists improved the GPs’ ability to recognise common mental disorders. This finding is consistent with our theory of change seeing the collaborative model as a complex intervention [26]. In a qualitative study of their experiences with our collaborative care model, GPs in the intervention practices emphasised the importance of co-location with mental health specialists for their learning and for their assessment and management of mental health problems [27]. Having opportunities for brief informal discussions with a co-located mental health specialist about a variety of clinical issues as they arise is an efficient way for GPs to acquire knowledge and skills [15, 27].

The increased recognition of anxiety, depression and common mental disorders in the intervention practices aligns with the results of a training programme in another CRCT [13]. In that study, patients with higher distress scores on a questionnaire were more likely to be recognized as having mental health problems, similar to the results in a study of our 2015 cohort [8]. Another study found that patients’ anxiety or depression was less likely to be recognized when GPs were less confident in their abilities to identify depression [14]. The validity of GPs’ diagnostic assessments could not be determined in our study due to lack of available data.

The results may also have been influenced by other factors than an increased recognition of mental disorders. The validity of the coding in our study is unknown as we had no other diagnostic resources to compare with. However, another Norwegian study has shown that GP’s ICPC-2 codes were consistent with their written notes in the patient records, indicating that codes may be expected to reflect the diagnostic work done by GPs [41].

The finding that an increased use of codes for common mental disorders was significantly associated with a reduction in codes for unexplained physical symptoms may indicate an increased awareness of common mental disorders and a lower threshold for using diagnostic codes for these. We see this as a positive result of the intervention, because an increased awareness of mental disorders was defined as a key element in the mechanism for change, when considering collaborative care as a complex intervention [26].

In the 2015 cohort, the intervention practices had lower recognition rates for anxiety and depression than the control practices, but we have no specific information on the GP groups which might indicate the reasons for this difference. However, we cannot rule out that regression to the mean may contribute to the results in our difference-in-difference analyses.

### GPs’ prescription of psychotropic medication

The intervention practices had a significantly higher increase in odds for prescribing antipsychotics, anxiolytics, and stimulants than the control practices. For prescribing hypnotics/sedatives, the intervention group had a reduction in odds while the control group had no change in odds, and the reduction in odds was significantly lower in the intervention group. When it came to prescribing antidepressants, both groups had a significant reduction in odds with no significant difference between groups.

We have found very little research on GPs’ prescriptions of various types of psychotropic medication. A study on utilisation of psychotropic medication in the mental health services in Norway over a decade showed that from 2015 to 2017 there was an increase in prescriptions of anxiolytics and stimulants, while there was a reduction in antidepressant prescriptions but little change in antipsychotics [42]. In a Swedish study, GPs were satisfied with their level of prescribing antidepressants and sedatives in relation to patient needs, but they felt they under-prescribed antipsychotics [43]. The results in these two studies seems to be consistent with some of the results in our study. One plausible interpretation for our results may be that close collaboration with co-located psychologists and psychiatrist enabled the GPs to initiate more diagnosis-specific psychopharmacological treatments (like with antipsychotics or stimulants) that are often started in mental health services following referral. The concurrent reduction in hypnotics/sedatives prescribing may indicate a shift from nonspecific symptom relief towards more targeted treatment of the underlying mental disorder. These interpretations are speculative and cannot be confirmed with the available data. Furthermore, because we lack access to clinical measurements we cannot know whether these changes are related to any improvement in clinical outcomes.

### The model and methodological issues

The two key positive results discussed above were GPs’ improved recognition of common mental disorders and a possibly more appropriate prescribing of psychotropic medication. This indicates that the collaborative care model, with close collaboration between co-located GPs and mental health specialists, may improve both the assessment and the management of common mental health disorders and problems. The model was also experienced as being time efficient for both patients and health professionals, in part because it led to earlier identification and initiation of treatment for emerging mental health problems [27].

Our collaborative care model focuses on co-location and close collaboration between GPs and mental health professionals, with both contributing complimentary competencies that are not limited to specific diagnoses or treatments, but which can be applied and adjusted to each individual patient or situation. The model is explained and discussed in more detail in a previous paper [26]. This model differs from many established collaborative care models which focus on specific and mostly chronic patient groups and programs, with specific components for each patient group [16, 19, 44].

One possible advantage with our model is that it is flexible and can be adapted for any patient or situation, similar to the HFHT model in Hamilton, Canada [15, 45]. The close collaboration also increases the knowledge and skills of both GPs and mental health specialists and broadens their understanding of ways in which they can use and support each other. While their collaboration is limited by their combined knowledge and skills, they both also have access to other colleagues and advisors who can be consulted when needed. With the GP practices being able to find space and supports for the mental health specialists, the GPs in our study found the model to be acceptable, feasible and helpful [26, 27].

The costs of our model will in part depend on adjustments for the time spent on the co-located collaboration, and especially how much treatment the mental health specialists themselves provide within the GP practices. An earlier model employed by the CMHC, which saw mental health specialists visiting GP practices once or twice a month, required less resources. This also aligns with a sustainable model practised by the first author as a psychiatrist visiting 20 GPs and other primary care providers each month across six municipalities in a CMHC catchment area, for one full day every week over 15 years [46]. Further discussions on other ways to make the model less resource-intensive and more sustainable and scalable are found in a previous paper on implementation of the model [26]. The division of collaborative care costs between GP practices and mental health services will depend on the funding system and financial support for collaborative care in the health care systems in each specific country or jurisdiction. This is discussed in several articles [47–49].

During 2015–2018, pressures within the Norwegian GP system became increasingly evident, including rising workloads and growing time constraints for GPs. Although this period prompted assessments, debate and early proposals for change, no reforms were implemented during the implementation of our study [50]. The increasing workloads and growing time constraints for GPs were similar for both intervention and control practices and were unlikely to have contributed to between-group differences in changes.

The randomisation resulted in the control group being twice as large as the intervention group, for both GPs and patients. Lack of data on the differences in GPs’ characteristics such as training background or prior experiences with collaboration limits the possibility of analysing whether such differences might have influenced the results. A review regarding the sizes of GP practices found limited evidence to support an association between practice size and quality of care in primary care [51]. However, we cannot exclude the possibility that such differences influenced the results in our study. The between-group differences in age and sex are significant but very small, with the significance likely achieved because of very large samples.

Longer term impacts on sickness leaves from work were analysed in another paper from our trial [28]. Fewer patients in the intervention group were on a sickness leave, but there was a 4% increase in total sick days due to longer absence durations and a less frequent use of graded sick leave. However, work assessment allowance days (long term sickness benefits) decreased by 8%. These changes were primarily among those with mental disorders and overall, this represents a significant socio-economic benefit. A Norwegian CRCT of a structured cognitive-behavioural communication tool for GPs treating patients with medically unexplained physical symptoms found similar results with a 27% points decrease in sickness leaves in the intervention group, which was significantly larger than the 4% point decrease in the control group [52]. If Norwegian GPs can improve their treatment with psychotropic medication and also provide cognitive behavioural therapy [53, 54], they may have two important skills for treatment of common mental disorders at their disposal.

### Strengths and limitations

Strengths of the study included the cluster-randomised controlled trial design, cohorts that included all patients who had contact with their GP over two 12-month periods, and large sample sizes. Limitations included the unknown validity of the GP’s diagnostic assessments, and the fact that the randomisation resulted in a control group that was twice as large as the intervention group, due to differences in the number of GPs in each practice. These factors may have influenced the results. In addition, the study was based on repeated cross-sectional samples and thus does not track within-person changes. Longitudinal follow-up would be needed to assess individual trajectories. Furthermore, no adjustment was made for multiple comparisons, which may increase the risk of false-positive findings. To address this, we have explicitly identified post hoc analyses and reported all results transparently so that readers can judge their robustness. Moreover, because the number of participating GP practices was small, potential baseline differences between practices may have influenced the results of the generalized linear mixed models, particularly for interaction effects. Consequently, these findings should be interpreted with caution. The variables that were available were limited to structured data on elements of health care, that were intermediate outcomes available in the electronic patient records, but which contained no structured measurements of clinical outcomes.

The trial registration was done retrospectively after the conclusion of the intervention but before the extracted data was available for analyses. The three boroughs also have worse socioeconomic conditions and a higher proportion of inhabitants with non-Norwegian ethnic backgrounds when compared to the rest of Oslo and our country. Availability sampling and small geographic areas may limit the generalisability to other places.

### Conclusions and implications

There were no significant differences in referrals to mental health services (primary outcome) and in the use of outpatient visits in mental health services. The GPs in the intervention practices diagnosed significantly more patients with common mental disorders (anxiety, depression), and these changes were significantly associated with a reduction in diagnoses for unexplained physical symptoms. Significant changes in prescribing patterns of psychotropic medication were consistent with the increased recognition of mental disorders, and their use was possibly more appropriate.

Co-located mental health specialists and GPs working together in GP practices may enhance mental health care in GP practices, lead to earlier recognition and treatment of mental health problems, and reduce prolonged illnesses with potential reductions in the need for long term sickness or disability benefits. While intermediate outcomes like recognition and prescriptions were changed, we cannot draw any conclusions regarding the effect on clinical outcomes, as the available data did not include measurements of clinical outcomes.

## Supplementary Information

Supplementary Material 1.

## Data Availability

The datasets used and analysed during the current study are available from the corresponding author on reasonable request.

## Abbreviations

ATC: Anatomical Therapeutic Chemical Classification System
CAMHS: Child and adolescent mental health services
CMHC: Community mental health centre
GP: General practitioner
INT: Intervention
CON: Control

## References

[1] Lampe L, Shadbolt N, Starcevic V, Boyce P, Brakoulias V, Hitching R, et al. Diagnostic processes in mental health: GPs and psychiatrists reading from the same book but on a different page. Australas Psychiatry. 2012;20(5):374–8. 23014118 10.1177/1039856212458007

[2] Klimas J, Neary A, McNicholas C, Meagher D, Cullen W. The prevalence of common mental and substance use disorders in general practice: a literature review and discussion paper. Mental Health Subst Use. 2014;7(4):497–508.

[3] Sundquist J, Ohlsson H, Sundquist K, Kendler KS. Common adult psychiatric disorders in Swedish primary care where most mental health patients are treated. BMC Psychiatry. 2017;17(1):235. 28666429 10.1186/s12888-017-1381-4 PMC5493066

[4] Sandvik H, Hetlevik O, Blinkenberg J, Hunskaar S. Continuity in general practice as predictor of mortality, acute hospitalisation, and use of out-of-hours care: a registry-based observational study in Norway. Br J Gen Pract. 2022;72(715):e84–90. 34607797 10.3399/BJGP.2021.0340 PMC8510690

[5] Kjosavik SR, Ruths S, Hunskaar S. Psychotropic drug use in the Norwegian general population in 2005: data from the Norwegian Prescription Database. Pharmacoepidemiol Drug Saf. 2009;18(7):572–8. 19402032 10.1002/pds.1756

[6] Kjosavik SR, Hunskaar S, Aarsland D, Ruths S. Initial prescription of antipsychotics and antidepressants in general practice and specialist care in Norway. Acta Psychiatr Scand. 2011;123(6):459–65. 21401533 10.1111/j.1600-0447.2011.01697.x

[7] Johnsen TM, Norberg BL, Krogh FH, Sigurdsson JA, Getz L. Complex issues in general practice – a prevalence study [Komplekse problemstillinger i allmennpraksis – en prevalensstudie]. Tidsskrift Den Norske legeforening. 2020;140(10):1017–22. 10.4045/tidsskr.19.0683 32602308

[8] Dahli MP, Haavet OR, Ruud T, Brekke M. GPs’ identification of patients with mental distress: a coupled questionnaire and cohort study from norwegian urban general practice. BMC Prim Care. 2022;23(1):260. 36210430 10.1186/s12875-022-01865-x PMC9549632

[9] Dahli MP, Saltyte-Benth J, Haavet OR, Ruud T, Brekke M. Somatic symptoms and associations with common psychological diagnoses: a retrospective cohort study from Norwegian urban general practice. Fam Pract. 2021;38(6):766–72. 34196347 10.1093/fampra/cmab038

[10] Haavet OR, Sirpal MK, Haugen W, Christensen KS. Diagnosis of depressed young people in primary health care–a validation of HSCL-10. Fam Pract. 2011;28(2):233–7. 20937663 10.1093/fampra/cmq078

[11] Tylee A, Ghandi P. The Importance of Somatic Symptoms in Depression in Primary Care. Prim Care Companion J Clin Psychiatry. 2005;7:167–76. 16163400 10.4088/pcc.v07n0405 PMC1192435

[12] Dahli MP, Brekke M, Ruud T, Haavet OR. Prevalence and distribution of psychological diagnoses and related frequency of consultations in Norwegian urban general practice. Scand J Prim Health Care. 2020;38(2):124–31. 10.1080/02813432.2020.1783477 PMC8570762 32594819

[13] Sinnema H, Majo MC, Volker D, Hoogendoorn A, Terluin B, Wensing M, et al. Effectiveness of a tailored implementation programme to improve recognition, diagnosis and treatment of anxiety and depression in general practice: a cluster randomised controlled trial. Implement Sci. 2015;10:33. 25884819 10.1186/s13012-015-0210-8 PMC4360947

[14] Sinnema H, Terluin B, Volker D, Wensing M, van Balkom A. Factors contributing to the recognition of anxiety and depression in general practice. BMC Fam Pract. 2018;19(1):99. 29935537 10.1186/s12875-018-0784-8 PMC6015659

[15] Kates N, McPherson-Doe C, George L. Integrating mental health services within primary care settings: the Hamilton Family Health Team. J Ambul Care Manag. 2011b;34(2):174–82. 10.1097/JAC.0b013e31820f6435 21415615

[16] Carron T, Rawlinson C, Arditi C, Cohidon C, Hong QN, Pluye P, et al. An Overview of Reviews on Interprofessional Collaboration in Primary Care: Effectiveness. Int J Integr Care. 2021;21(2):31. 34220395 10.5334/ijic.5588 PMC8231476

[17] Harkness EF, Bower PJ. On-site mental health workers delivering psychological therapy and psychosocial interventions to patients in primary care: effects on the professional practice of primary care providers. Cochrane Database of Systematic Reviews. Issue 1. Art. No.: CD000532.pub2. 10.1002/14651858.CD000532. 10.1002/14651858.CD000532.pub2 PMC7068168 19160181

[18] Isaacs AN, Mitchell EKL. Mental health integrated care models in primary care and factors that contribute to their effective implementation: a scoping review. Int J Ment Health Syst. 2024;18(1):5. 38331913 10.1186/s13033-024-00625-x PMC10854062

[19] Woltmann E, Grogan-Kaylor A, Perron B, Georges H, Kilbourne AM, Bauer MS. Comparative effectiveness of Collaborative Chronic Care Models for Mental Health Conditions Across Primary, Specialty, and Behavioral Health Care Settings: Systematic Review and Meta-Analysis. Am J Psychiatry. 2012;169(8):790–804. 22772364 10.1176/appi.ajp.2012.11111616

[20] Archer J, Bower P, Gilbody S, Lovell K, Richards D, Gask L, et al. Collaborative care for depression and anxiety problems. Cochrane Database Syst Rev. 2012;10(10):CD006525. 23076925 10.1002/14651858.CD006525.pub2 PMC11627142

[21] Woods JB, Greenfield G, Majeed A, Hayhoe B. Clinical effectiveness and cost effectiveness of individual mental health workers colocated within primary care practices: a systematic literature review. BMJ Open. 2020;10(12):e042052. 33268432 10.1136/bmjopen-2020-042052 PMC7713190

[22] Gilbody S, Bower P, Fletcher J, Richards D, Sutton AJ. Collaborative Care for Depression. A Cumulative Meta-analysis and Review of Longer-term Outcomes. Arch Intern Med. 2006;166:2314–21. 17130383 10.1001/archinte.166.21.2314

[23] Kates N. Integrating Mental Health Services: Principles, Practices and Possibilities. Chapter 4, pages 1-16. In: In Primary Care Medicine - Theory and Practice, edited by H. Cakmur. London. UK Intechopen; 2023. 10.5772/intechopen.1002786.

[24] Kates N, Arroll B, Currie E, Hanlon C, Gask L, Klasen H, et al. Improving collaboration between primary care and mental health services. World J Biol Psychiatry. 2019;20(10):748–65. 29722600 10.1080/15622975.2018.1471218

[25] Kates N, Mazowita G, Lemire F, Jayabarathan A, Bland R, Selby P, et al. The Evolution of Collaborative Mental Health Care in Canada: A Shared Vision for the Future. Position Paper. Can J Psychiatry. 2011;56(5):1–10.

[26] Ruud T, Rugkåsa J, Haavet OR, Dahli MP, Hanssen-Bauer K, Brekke M, et al. A collaborative primary and mental health care model with psychologist and psychiatrist working in GP practices: process evaluation of the implementation, challenges, and sustainability. BMC Health Serv Res. 2025;25(1):1178. 10.1186/s12913-025-13408-y. 10.1186/s12913-025-13408-y PMC12403931 40898247

[27] Rugkasa J, Tveit OG, Berteig J, Hussain A, Ruud T. Collaborative care for mental health: a qualitative study of the experiences of patients and health professionals. BMC Health Serv Res. 2020;20(1):844. 32907559 10.1186/s12913-020-05691-8 PMC7487713

[28] Kann IC, Dahl MP, Ruud T. Ny samhandlingsmodell for pasienter med psykiske lidelser: Påvirkes sykefravær eller arbeidsavklaringspenger? [New collaborative care model for patients with mental disorders: Is sick leave or work assessment allowance money affected?]. Arbeid og velferd. 2019;(2):27–41. https://arbeidogvelferd.nav.no/journal/2019/2.

[29] Haavet OR, Saltyte Benth J, Gjelstad S, Hanssen-Bauer K, Dahli MP, Kates N, et al. Detecting young people with mental disorders: a cluster-randomised trial of multidisciplinary health teams at the GP office. BMJ Open. 2021;11(12):e050036. 34952870 10.1136/bmjopen-2021-050036 PMC8712985

[30] Schulz KF, Altman DG, Moher D, Group C. CONSORT 2010 Statement: updated guidelines for reporting parallel group randomised trials. BMC Med. 2010;8:18. 20334633 10.1186/1741-7015-8-18 PMC2860339

[31] Moher D, Hopewell S, Schulz KF, Montori V, Gotzsche PC, Devereaux PJ, et al. CONSORT 2010 Explanation and Elaboration: Updated guidelines for reporting parallel group randomised trials. J Clin Epidemiol. 2010;63(8):e1–37. 20346624 10.1016/j.jclinepi.2010.03.004

[32] OECD. OECD Reviews of Health Care Quality: Norway 2014: Raising Standards. Mental health in Norway. 2014. 10.1787/9789264208469-en.

[33] Ringard Å, Sagan A, Perre Saunes I, Lindahl AK, Norway. Health system review. Copenhagen: European Observatory on Health Systems and Policies; 2013. p. 8. Contract No. 24434287

[34] Allmennlegeforeningen. Innbyggere uten fastlege [Inhabitants without a regular general practitioner]: Den norske legeforening [The Norwegian Medical Association]; 2025 [Available from: https://www.legeforeningen.no/foreningsledd/yf/allmennlegeforeningen/krisen-i-fastlegeordningen/lister-uten-fast-lege/

[35] Tikkanen R, Osborn R, Mossialos E, Djordjevic A, Wharton GA. International Health Care System Profiles. New York: Norway; 2020.

[36] Proctor E, Silmere H, Raghavan R, Hovmand P, Aarons G, Bunger A, et al. Outcomes for implementation research: conceptual distinctions, measurement challenges, and research agenda. Adm Policy Ment Health. 2011;38(2):65–76. 20957426 10.1007/s10488-010-0319-7 PMC3068522

[37] WONCA. International Classification of Primary Care. 2nd ed. revised (ICPC-2-R). England: Oxford University Press; 2005. 10.1093/oso/9780198568575.001.0001.

[38] WHO. The Anatomical Therapeutic Chemical Classification System with Defined Daily Doses (ATC/DDD). World Health Organisation; [Available from: https://www.who.int/standards/classifications/other-classifications/the-anatomical-therapeutic-chemical-classification-system-with-defined-daily-doses

[39] Ruths S, Haukenes I, Hetlevik O, Smith-Sivertsen T, Hjorleifsson S, Hansen AB, et al. Trends in treatment for patients with depression in general practice in Norway, 2009–2015: nationwide registry-based cohort study (The Norwegian GP-DEP Study). BMC Health Serv Res. 2021;21(1):697. 34266438 10.1186/s12913-021-06712-w PMC8283991

[40] Tveit OG, Ruud T, Hanssen-Bauer K, Haavet OR, Hussain A. An explorative study of factors associated with treatment in specialized mental health care centers among GP patients in Norway. BMC Health Serv Res. 2021;21(1):960. 34517868 10.1186/s12913-021-06982-4 PMC8436443

[41] Sporaland GL, Mouland G, Bratland B, Rygh E, Reiso H. General practitioners’ use of ICPC diagnoses and their correspondence with patient record notes. Tidsskrift Den Norske legeforening. 2019;139(15):1–9. 10.4045/tidsskr.18.0440 31642635

[42] Seljeflot LM, Blix HS, Andersson Y, Hynnekleiv T. Forbruk av psykofarmaka i spesialisthelsetjenesten for psykisk helsevern og rusbehandling 2012–23 [Consumption of psychotropic drugs in specialist mental health and substance abuse treatment services 2012–23]. Tidsskrift Den Norske legeforening. 2025;145(6). 10.4045/tidsskr.24.0531. 10.4045/tidsskr.24.0531 40366037

[43] Svensson SA, Hedenrud TM, Wallerstedt SM. Attitudes and behaviour towards psychotropic drug prescribing in Swedish primary care: a questionnaire study. BMC Fam Pract. 2019;20(1):4. 30611211 10.1186/s12875-018-0885-4 PMC6321667

[44] Miller CJ, Grogan-Kaylor A, Perron BE, Kilbourne AM, Woltmann E, Bauer MS. Collaborative chronic care models for mental health conditions: cumulative meta-analysis and metaregression to guide future research and implementation. Med Care. 2013;51(10):922–30. 23938600 10.1097/MLR.0b013e3182a3e4c4 PMC3800198

[45] Kates N, Ng V, Sunderji N, Patriquin M, Alloo J, Mirwaldt P, et al. Collaborative Mental Health Care in Canada: Challenges, Opportunities and New Directions. Can J Psychiatry. 2023;68(5):372–98. 36688252 10.1177/07067437221102201 PMC10192825

[46] Ruud T, Aarre TF, Boeskov B, Husevåg PS, Klepp R, Kristiansen SA, et al. Satisfaction with primary care and mental health care among individuals with severe mental illness in a rural area: a seven-year follow-up study of a clinical cohort. Int J Ment Health Syst. 2016;10:33. 27073414 10.1186/s13033-016-0064-8 PMC4828811

[47] Grazier KL, Smiley ML, Bondalapati KS. Overcoming Barriers to Integrating Behavioral Health and Primary Care Services. J Prim Care Community Health. 2016;7(4):242–8. 10.1177/2150131916656455 PMC5932707 27380923

[48] O’Donnell AN, Williams M, Kilbourne AM. Overcoming roadblocks: current and emerging reimbursement strategies for integrated mental health services in primary care. J Gen Intern Med. 2013;28(12):1667–72. 23733375 10.1007/s11606-013-2496-z PMC3832738

[49] Ross KM, Gilchrist EC, Melek SP, Gordon PD, Ruland SL, Miller BF. Cost savings associated with an alternative payment model for integrating behavioral health in primary care. Transl Behav Med. 2019;9(2):274–81. 29796605 10.1093/tbm/iby054

[50] Holden MB, Rasmussen I. Evaluering av fastlegeordningen [Evaluation of the GP scheme]. Oslo: EY og Vista Analyse; 2019.

[51] Ng CW, Ng KP. Does practice size matter? Review of effects on quality of care in primary care. Br J Gen Pract. 2013;63(614):e604–10. 23998840 10.3399/bjgp13X671588 PMC3750799

[52] Abrahamsen C, Reme SE, Wangen KR, Lindbaek M, Werner EL. The effects of a structured communication tool in patients with medically unexplained physical symptoms: a cluster randomized trial. EClinicalMedicine. 2023;65:102262. 37855023 10.1016/j.eclinm.2023.102262 PMC10579279

[53] Haavet OR, Myhrer KS, Kates N, Mdala I, Aschim B, Lundevall S, et al. Impact of a cognitive behavioural therapy training program on family physician practices Cross-sectional study in Norway. Can Fam Physician. 2023;69:784–91. 10.46747/cfp.6911784 PMC10645441 37963798

[54] Truelove S, Ng V, Kates N, Alloo J, Sunderji N, Patriquin MJ. Collaborative mental health care: Engaging health systems to support a team-based approach. Can Fam Physician. 2023;69(2):81–3. 10.46747/cfp.690281 PMC9945898 36813511

