# Supplementary material for: Effects of collaborative care on recognition and management of common mental disorders by general practitioners: a cluster-randomised trial in Norway
Source: BMC Prim Care. 2026 Feb 26;27:105. doi: 10.1186/s12875-026-03227-3 (PMC13020071; doi:10.1186/s12875-026-03227-3)
Supplement: Supplementary file 1 — Supplementary Material 1. [file 12875_2026_3227_MOESM1_ESM.pdf]

Supplementary material

Page 1

We have included a figure illustrating the design of the cluster-randomised controlled trial to show the relationships between cohorts (2015, 2017) and groups (intervention and control groups).

Pages 2-3

The supplementary material tables showing additional information of the results from the generalized linear mixed models. Here the results have been transferred to odds ratios, which may be more accessible for many readers and give the same information as line diagrams.

Figure 1. The design of the cluster-randomised controlled trial.

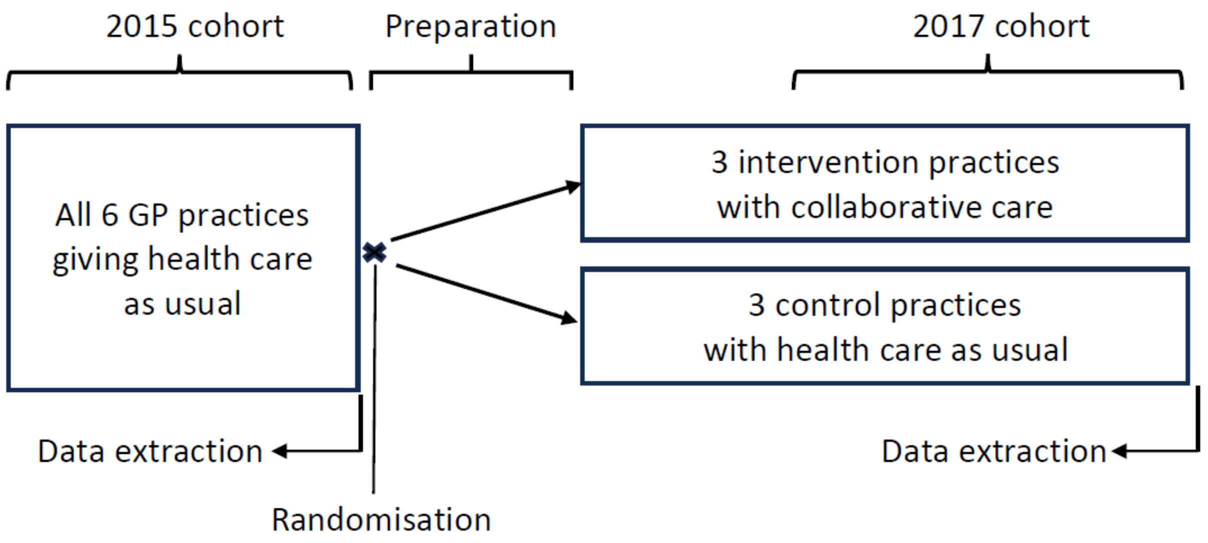

Supplementary Table 2B on referrals.

Results of generalized linear mixed model of referrals to mental health services overall (OR=odds ratio, CI=confidence interval)

	Intervention practices	Control practices	Intervention vs control practices	
**Cohort**	**OR* (95% CI)**	**OR* (95% CI)**	**OR* (95% CI)**	**p-value**
2015	0.001 (0.0008;0.002)	0.002 (0.001;0.002)	0.69 (0.51;0.94)	**0.018**
2017	0.001 (0.0008;0.002)	0.001 (0.0009;0.002)	0.90 80.66;1.21)	0.485
2015 vs. 2017	1.04 (0.75;1.43)	0.80 (0.65;0.98)	1.30 (0.89; 1.91)	0.174

* Very low odds are due to sparse data in some groups, resulting in extreme but valid point estimates.

Supplementary Table 3B on outpatients in mental health services.

Results of generalized linear mixed models for GP patients being outpatients in mental health services (OR=odds ratio, CI=confidence interval)

	Intervention practices	Control practices	Intervention vs control practices	
**Cohort**	**OR* (95% CI)**	**OR* (95% CI)**	**OR* (95% CI)**	**p-value**
**Adolescents**				
2015	0.33 (0.0004;256.5)	0.27 (0.0003;205.8)	1.24 (0.67;2.30)	0.847
2017	0.35 (0.0004;275.9)	0.31 (0.0004;235.1)	1.15 (0.66;1.98)	0.598
2015 vs. 2017	1.07 (0.56;2.03)	1.15 (0.68;1.93)	0.93 (0.41; 2.11)	0.856
**Adults**				
2015	0.01 (0.006;0.01)	0.01 (0.01;0.02)	0.71 (0.55;0.91)	**.008**
2017	0.01 (0.004;0.01)	0.01 (0.01;0.01)	0.61 (0.47;0.79)	**<0.001**
2015 vs. 2017	0.71 (0.54;0.94)	0.83 (0.70;0.98)	0.86 (0.62; 1.19)	0.363

Results of generalized linear mixed models of average number of outpatient visits for GP patients' being outpatients in mental health services (CI=confidence interval)

	Intervention practices	Control practices	Intervention vs control practices	
**Cohort**	**Mean (95% CI)**	**Mean (95% CI)**	**Mean difference (95% CI)**	**p-value**
**Adolescents**				
2015	36.3 (-14.6;87.2)	39.0 (-11.6;89.7)	-2.8 (-7.4;1.9)	0.098
2017	40.4 (-11.3;92.0)	38.2 (-12.8;89.1)	2.2 (-2.0;6.4)	0.662
2015 vs. 2017	4.1 (-0.8;8.9)	-0.9 (-4.8;3.1)	5.0 (-1.2; 11.2)	0.117
**Adults**				
2015	12.4 (9.3;15.6)	13.1 (10.3;15.8)	-0.6 (-2.9;1.7)	0.601
2017	13.3 (10.2;16.4)	13.0 (10.3;15.7)	0.3 (-2.2;2.7)	0.823
2015 vs. 2017	0.8 (-1.9;3.6)	-0.05 (-1.7;1.6)	0.9 (-2.3; 4.1)	0.589

* Very low odds are due to sparse data in some groups, resulting in extreme but valid point estimates.

Supplementary Table 4B on GPs' recognition of patients with common mental disorders, anxiety and depression in intervention and control GP practices at least once for the 2015 and 2017 cohorts.

Results of generalized linear mixed models for common mental disorders, anxiety and depression (OR=odds ratio, CI=confidence interval)

	Intervention practices	Control practices	Intervention vs control practices	
**Problems / Cohort**	**OR* (95% CI)**	**OR* (95% CI)**	**OR* (95% CI)**	**p-value**
**Common mental disorder**				
2015	0.05 (0.04;0.06)	0.07 (0.06;0.08)	0.70 (0.61;0.80)	**<0.001**
2017	0.06 (0.05;0.07)	0.06 (0.05;0.07)	0.95 (0.84;1.08)	0.460
2015 vs. 2017	1.24 (1.07;1.45)	0.91 (0.83;1.01)	1.36 (1.14;1.64)	**0.001**
**Anxiety**				
2015	0.002 (0.001;0.003)	0.002 (0.001;0.003)	0.71 (0.52;0.97)	**0.030**
2017	0.002 (0.001;0.004)	0.002 (0.001;0.003)	1.32 (0.99;1.76)	0.062
2015 vs. 2017	1.53 (1.12;2.09)	0.83 (0.67;1.02)	1.85 (1.26;2.70)	**0.001**
**Depression**				
2015	0.003 (0.002;0.004)	0.005 (0.003;0.007)	0.54 (0.43;0.68)	**<0.001**
2017	0.004 (0.002;0.005)	0.004 (0.003;0.006)	0.81 (0.65;1.00)	0.055
2015 vs. 2017	1.30 (1.03;1.64)	0.88 (0.76;1.01)	1.48 (1.13;1.96)	**0.005**

* Very low odds are due to sparse data in some groups, resulting in extreme but valid point estimates.

Supplementary Table 5B on prescription of different types of psychotropic drugs in GP intervention and control practices at least once for 2015 and 2017 cohorts. Results of linear mixed models for types of psychotropic medication (OR=odds ratio, CI=confidence interval)

	Intervention practices	Control practices	Intervention vs control practices	
**Type / Cohort**	**OR* (95% CI)**	**OR* (95% CI)**	**OR* (95% CI)**	**p-value**
**Antipsychotics**				
2015	0.006 (0.004;0.009)	0.008 (0.006;0.01)	0.68 (0.49;0.94)	**0.021**
2017	0.008 (0.005;0.01)	0.008 (0.006;0.01)	1.06 (0.77;1.46)	0.712
2015 vs. 2017	1.45 (1.12;1.87)	0.93 (0.81;1.07)	1.56 (1.16;2.09)	**0.003**
**Anxiolytics**				
2015	0.02 (0.01;0.03)	0.02 (0.01;0.04)	0.81 (0.62;1.06)	0.124
2017	0.02 (0.01;0.04)	0.02 (0.01;0.04)	1.07 (0.82;1.40)	0.604
2015 vs. 2017	1.20 (0.99;1.45)	0.90 (0.81;1.01)	1.32 (1.06;1.65)	**0.012**
**Hypnotics/Sedatives**				
2015	0.08 (0.05;0.13)	0.08 (0.05;0.12)	1.00 (0.77;1.29)	0.988
2017	0.06 (0.03;0.10)	0.08 (0.05;0.13)	0.69 (0.53;0.90)	**0.005**
2015 vs. 2017	0.75 (0.63;0.89)	1.08 (0.98;1.20)	0.69 (0.57;0.84)	**<0.001**
**Antidepressants**				
2015	0.33 (0.20;0.53)	0.23 (0.15;0.36)	1.43 (1.11;1.84)	**0.006**
2017	0.29 (0.18;0.48)	0.23 (0.14;0.35)	1.30 (1.00;1.68)	**0.047**
2015 vs. 2017	0.89 (0.74;1.08)	0.98 (0.87;1.10)	0.91 (0.73;1.13)	0.398
**Stimulants**				
2015	0.51 (0.32;0.80)	0.95 (0.67;1.35)	0.54 (0.37;0.77)	**0.001**
2017	1.34 (0.93;1.93)	1.52 (1.09;2.11)	0.88 (0.69;1.13)	0.321
2015 vs. 2017	2.63 (1.77;3.93)	1.60 (1.32;1.94)	1.65 (1.06;2.56)	**0.027**

* Very low odds are due to sparse data in some groups, resulting in extreme but valid point estimates.
